# GDF-15 Levels and Other Laboratory Findings as Predictors of COVID-19 Severity and Mortality: A Pilot Study

**DOI:** 10.3390/biomedicines12040757

**Published:** 2024-03-29

**Authors:** Luka Švitek, Dubravka Lišnjić, Barbara Grubišić, Mihaela Zlosa, Ema Schönberger, Nika Vlahović Vlašić, Petra Smajić, Dario Sabadi, Tara Rolić, Kristina Kralik, Sanja Mandić

**Affiliations:** 1 Clinic for Infectious Diseases, University Hospital Centre Osijek, 31000 Osijek, Croatia; 2 Department of Infectology and Dermatovenerology, Faculty of Medicine Osijek, J. J. Strossmayer University of Osijek, 31000 Osijek, Croatia; 3 Faculty of Dental Medicine and Health Osijek, J. J. Strossmayer University of Osijek, 31000 Osijek, Croatia; 4 Faculty of Medicine Osijek, J. J. Strossmayer University of Osijek, 31000 Osijek, Croatia; 5 Department of Endocrinology, Internal Medicine Clinic, University Hospital Centre Osijek, 31000 Osijek, Croatia; 6 Institute of Clinical Laboratory Diagnostics, University Hospital Centre Osijek, 31000 Osijek, Croatia; 7 Department of Medical Statistics and Medical Informatics, Faculty of Medicine Osijek, J. J. Strossmayer University of Osijek, 31000 Osijek, Croatia; 8 Polyclinic LabPlus, 31000 Osijek, Croatia; 9 Department of Chemistry, Biochemistry and Clinical Chemistry, Faculty of Medicine Osijek, J. J. Strossmayer University of Osijek, 31000 Osijek, Croatia

**Keywords:** COVID-19, growth differentiation factor 15, inflammation, L-Lactate Dehydrogenase, mortality, SARS-CoV-2

## Abstract

Growth differentiation factor 15 (GDF-15) is a stress-induced cytokine associated with acute and chronic inflammatory states. This prospective observational study aimed to investigate the prognostic roles of GDF-15 and routine clinical laboratory parameters in COVID-19 patients. Upon the admission of 95 adult hospitalized COVID-19 patients in Croatia, blood analysis was performed, and medical data were collected. The patients were categorized based on survival, ICU admission, and hospitalization duration. Logistic regression and ROC curve methods were employed for the statistical analysis. Logistic regression revealed two independent predictors of negative outcomes: CURB-65 score (OR = 2.55) and LDH (OR = 1.005); one predictor of ICU admission: LDH (OR = 1.004); and one predictor of prolonged hospitalization: the need for a high-flow nasal cannula (HFNC) upon admission (OR = 4.75). The ROC curve showed diagnostic indicators of negative outcomes: age, CURB-65 score, LDH, and GDF-15. The largest area under the curve (AUC = 0.767, specificity = 65.6, sensitivity = 83.9) was represented by GDF-15, with a cutoff value of 3528 pg/mL. For ICU admission, significant diagnostic indicators were LDH, CRP, and IL-6. Significant diagnostic indicators of prolonged hospitalization were CK, GGT, and oxygenation with an HFNC upon admission. This study reaffirms the significance of the commonly used laboratory parameters and clinical scores in evaluating COVID-19. Additionally, it introduces the potential for a new diagnostic approach and research concerning GDF-15 levels in this widespread disease.

## 1. Introduction

### 1.1. GDF-15 Expression and Its Role as an Immune Mediator

Growth differentiation factor 15 is a member of the transforming growth factor-β (TGF-β) superfamily, expressed in the cells of reproductive organs such as the placenta and prostate. However, under stress conditions, its expression can be induced in endothelial cells, vascular smooth muscle cells, cardiomyocytes, adipocytes, and macrophages. Additionally, research indicates that local blood circulation obstruction can trigger the release of GDF-15 [1].

Under normal physiological conditions, the GDF-15 levels remain low in healthy individuals, in contrast to patients with certain malignant diseases, pregnant women, and obese individuals [1,2,3,4,5]. Moreover, GDF-15 expression increases with age and is associated with tissue stress and injury [1,6,7,8], as well as with chronic inflammatory conditions, such as rheumatoid arthritis, Behçet’s disease, and type I diabetes mellitus. Therefore, it can be assumed that not only acute but also chronic inflammation can lead to an increase in GDF-15 levels, likely due to its protective role in both cases [1,9,10,11]. 

Additionally, despite its presumed protective role in tissues, since it is typically triggered by more severe forms of underlying disease, GDF-15 is commonly identified as a biomarker associated with an unfavourable disease course. 

### 1.2. GDF-15 and COVID-19

As mentioned, GDF-15 is likely released from the endothelial cells. Given that SARS-CoV-2 is known for both direct and indirect endothelial damage, an elevation of GDF-15 in COVID-19 can be anticipated [12].

In two proteomics analyses, a positive correlation was observed between a higher GDF-15 expression, disease severity, and the duration of intensive care unit (ICU) recovery [13,14]. Additionally, there are indications that the GDF-15 levels could be higher in hospitalized SARS-CoV-2 patients than in discharged patients who do not require hospitalization [15,16] and also in patients with poorer respiratory function at hospital admission [17,18], as well as those with a worse clinical outcome [19,20,21]. Furthermore, a study on autopsy lungs and plasma samples correlated GDF-15 levels with tissue damage and fibrotic remodelling of the lungs in COVID-19 patients [22]. On the contrary, a study by Delaye et al. reported lower median levels of GDF-15 in COVID-19 patients compared to SARS-CoV-2-negative patients [23], and another study implied that there is no association between GDF-15 and the need for hospitalization in SARS-CoV-2 patients [24]. 

### 1.3. Study Design, Objectives, and Hypothesis

This prospective observational cohort pilot study, conducted at the University Hospital Centre Osijek, aimed to investigate the prognostic significance of GDF-15 in COVID-19 patients in relation to mortality, ICU admission, and length of hospitalization. Our hypothesis suggested that patients with higher initial levels of GDF-15 would be more likely to experience fatal outcomes, ICU admission, and extended hospitalization. Additionally, we evaluated the prognostic value of routine clinical laboratory parameters in this study. Ethical approval for this research was obtained from both the Ethics Committee of the University Hospital Centre Osijek and the Ethics Committee of the Faculty of Medicine Osijek.

To the best of our knowledge, this study is the first to investigate the correlation between GDF-15 expression and three crucial indicators for evaluating disease severity: clinical outcomes, ICU admission, and the length of hospitalization.

## 2. Materials and Methods

### 2.1. Participants

The participants of this prospective study were patients aged 18 years and older, initially hospitalized in the Clinic for Infectious Diseases at the University Hospital Centre Osijek during a 45-day period amid the COVID-19 pandemic. The study’s inclusion criteria involved hospitalization necessity and a positive SARS-CoV-2 polymerase chain reaction (PCR) test, with the sample obtained using a nasopharyngeal swab. Patients who had not survived 72 h after admission were excluded from the study. In total, 95 patients were enrolled in this study, and all the patients were followed up from hospital admission until discharge or a lethal outcome. Informed consent was obtained from the patients enrolled in the study or their legal guardians, and the research was conducted in accordance with the previously mentioned ethics committee approvals and the Declaration of Helsinki.

To detect differences in continuous variables with an effect size (f) of 0.25, a test power of 0.80, and a significance level of 0.05, the minimum sample size is determined to be 44 patients. Furthermore, for regression analysis with a test power of 80%, a minimum sample size of 56 participants is required. In conclusion, the established minimum sample size for the research was 56 patients (G*Power, 3.1.2).

### 2.2. Methods

Upon admission, a blood analysis was immediately performed on each patient enrolled in the study. The samples were obtained via venipuncture, and sampling was undertaken using a 4 mL BD Vacutainer^®^ CAT (Clot Activator Tube); a 3 mL BD Vacutainer^®^ K2EDTA Tube; and a 2.7 mL BD Vacutainer^®^ 9NC Trisodium Citrate Tube (Becton Dickinson and Company, Belliver Industrial Estate, Plymouth, UK). 

The analysis of the samples collected in the 3 mL BD Vacutainer^®^ K2EDTA for complete blood count (CBC) and differential blood count (DBC) was conducted using a hematology analyzer Sysmex XN-2000 (Sysmex Corporation, Kobe, Japan).

The sample collected in the 2,7 mL BD Vacutainer^®^ 9NC Trisodium Citrate Tube was centrifuged for 10 min at 2000× *g*, and subsequently, the prothrombin time (PT), D-dimers, fibrinogen (Fbg), antithrombin 3 (AT 3), and activated partial thromboplastin time (aPTT) were measured using a BCS XP coagulometer (Siemens Healthineers AG, Erlangen, Germany).

The sample from the 4 mL BD Vacutainer^®^ CAT (Clot Activator Tube) was centrifuged for 10 min at 2000× *g*. In the serum sample, urea (BUN), creatinine, aspartate aminotransferase (AST), alanine aminotransferase (ALT), lactate dehydrogenase (LDH), alkaline phosphatase (ALP), gamma-glutamyltransferase (GGT), creatine kinase (CK), and albumin were determined using the spectrophotometric method; C-reactive protein (CRP) and ferritin were measured using the immunoturbidimetric method; and sodium (Na), potassium (K), and chloride (Cl) were measured using indirect potentiometry, all using the AU480 analyzer (Beckman Coulter, Brea, CA, USA) and using the same reagents according to the manufacturer’s instructions.

Additionally, interleukin-6 (IL-6), procalcitonin (PCT), and growth differentiation factor 15 (GDF-15) were measured in the serum sample using an electrochemiluminescent method (ECLIA) using the COBAS e601 immunoassay analyzer (Roche Diagnostics GmbH, Mannheim, Germany), and high-sensitivity troponin I (hsTnI) was measured using a homogeneous immunoassay method based on LOCI technology using the Dimension EXL analyzer with LM (Siemens Healthcare Diagnostics, Newark, NJ, USA).

All the mandatory laboratory health and safety procedures were complied with during the course of conducting the experimental work reported in this paper.

Chest X-rays were performed upon admission to radiologically confirm or rule out pneumonia, to the extent permitted by this recording method.

In addition to the aforementioned measurements, patients’ data were collected from their medical history. The collected data included age, sex, vaccination information, information about earlier SARS-CoV-2 infection, comorbidities (diabetes mellitus, cardiac disease, lung disease, chronic kidney disease), clinical signs and symptoms of disease at admission, systolic and diastolic blood pressure, peripheral blood oxygen saturation, heart rate, respiratory rate, Glasgow Coma Scale (GCS) score, length of hospitalization, the need for ICU admission, and clinical outcomes.

Additionally, it was documented whether the patient required a high-flow nasal cannula (HFNC) and/or invasive mechanical ventilation (IMV) during hospitalization.

The CURB-65 score [25] (1 point for confusion, urea >7 mmol/L, respiratory rate ≥30, systolic blood pressure <90 mmHg or diastolic blood pressure ≤60 mmHg, age ≥65 years; maximum 5 points) and the quick Sequential Organ Failure Assessment (qSOFA) score [26] (1 point for altered mental status, GCS <15, respiratory rate ≥22, systolic blood pressure <100 mmHg; maximum 3 points) were calculated of each participant, as well as their mean arterial pressure (MAP). MAP was calculated using systolic and diastolic blood pressure: (systolic blood pressure + double diastolic blood pressure) divided by 3.

### 2.3. Statistical Methods

Categorical data were presented using absolute and relative frequencies. Differences in the categorical variables were assessed using the Chi-square test and Fisher’s exact test. The normality of the distribution of the continuous variables was tested using the Shapiro–Wilk test. Continuous data were described using the median and interquartile range. Differences in the continuous variables between two independent groups were examined using the Mann–Whitney U test. Correlation was evaluated using Spearman’s correlation coefficient (ρ). Logistic regression analysis (bivariate, multivariate—stepwise method) was used to analyze the independent factors associated with the outcomes. The receiver operating curve (ROC) was used to determine the optimal threshold, area under the curve (AUC), specificity, and sensitivity of the tested parameters. All the *p* values were two-tailed. The significance level was set at Alpha = 0.05. The statistical analyses were performed using MedCalc^®^ statistical software version 22.018 (MedCalc Software Ltd., Ostend, Belgium; https://www.medcalc.org; 2024, accessed on 8 January 2024) and SPSS version 23.0 (Released 2015. IBM. Armonk, NY, USA: IBM Corp.).

## 3. Results

After the exclusion of patients who did not meet the inclusion criteria or met exclusion criteria, our study comprised 95 participants. The baseline demographic and clinical characteristics of the participants are summarized in Table 1.

A total of 34 patients (36%) were vaccinated, and none of them had a prior history of COVID-19 infection. The most prevalent comorbidity among the cohort was arterial hypertension, followed by type 2 diabetes mellitus and cardiomyopathy (Table 1).

Concerning the clinical symptoms and signs, fever was the most frequently reported general symptom, followed by general weakness. Among the respiratory symptoms, cough was the most common, while dyspnea was reported during the examination in 77% of cases and in the anamnesis of 63% of patients. The majority of the patients presented with pneumonia, predominantly bilateral, necessitating oxygen therapy in most cases. Some individuals encountered complications, including pleural effusion, and required high-flow nasal cannula (HFNC) support, as detailed in Table 1.

**Figure 1:**
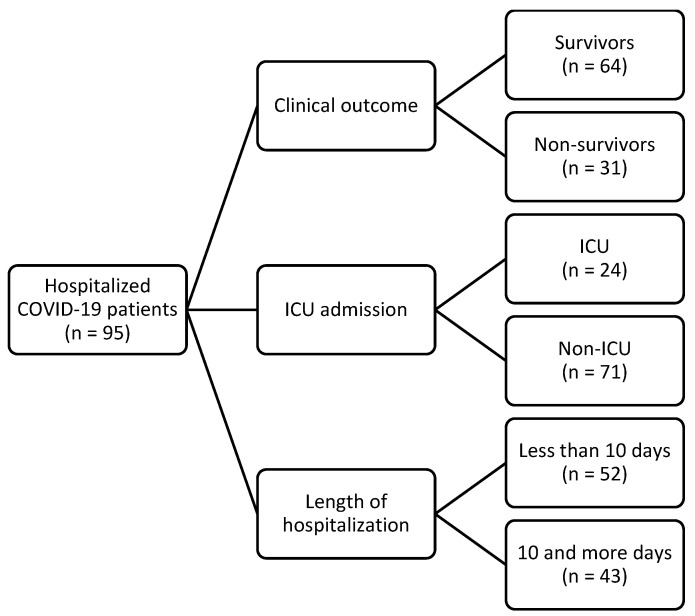
Flowchart of participants enrolled in this study. ICU—intensive care unit.

The patients were divided into groups, as shown in the figure (Figure 1).

Regarding the clinical outcomes, patients with a fatal outcome are significantly older and have a higher frequency of cardiomyopathy as a comorbidity. Additionally, their qSOFA score and CURB-65 score upon admission are significantly higher. In terms of the laboratory findings upon admission, patients with a fatal outcome, compared to those who survived, have significantly lower values of MCHC, platelets, and eosinophils. They also exhibit significantly higher values of RDW-CV, AST, CK, LDH, urea, creatinine, CRP, high-sensitivity troponin I, IL-6, and GDF-15 (Table 2).

Patients transferred to the ICU show significantly higher values of AST, LDH, and CRP compared to other patients (Table 2).

Patients hospitalized for 10 days or more exhibit significantly higher levels of GGT, CK, LDH, and CRP upon admission, in contrast to those with a hospitalization period of up to 10 days (Table 2).

We used Spearman’s correlation coefficient (Rho) to examine the association of GDF-15 with age and the qSOFA and CURB-65 scores, as well as the values of inflammatory markers (CRP, PCT, IL-6) upon admission, considering the outcome and transfer to the ICU. In the group of subjects with a negative outcome, a positive and significant association was found between GDF-15 and the qSOFA score, as well as with the values of PCT upon admission. For patients transferred to the ICU, an association was observed between GDF-15 and PCT upon admission (Table 3).

Logistic regression was conducted to investigate the influence of multiple factors on the likelihood of a negative treatment outcome. Independent predictors are variables that exhibited a change concerning the treatment outcome. The individual impact of each predictor on a negative outcome is detailed in Table 4. The model considered predictors that were significant in the bivariate analysis. Comprising two predictors, the model is entirely statistically significant (χ^2^ = 23.7, *p* < 0.001) and explains between 23% (according to Cox and Snell’s method) and 32% (according to Nagelkerke’s method) of the variance in the presence of a negative outcome, accurately classifying 79% of cases. Only two independent predictors made a unique statistically significant contribution to the model: CURB-65 score and LDH upon admission. The stronger predictor is the CURB-65 score, meaning that patients with higher score values have a 2.55 times greater chance of a fatal outcome (Table 4).

The independent predictors significant in predicting the probability of transfer to the ICU are presented in Table 5. Through multivariate logistic regression, one predictor stood out, LDH upon admission, which was entirely statistically significant (χ^2^ = 5.18, *p* = 0.02), explaining between 5% (according to Cox and Snell’s method) and 8% (according to Nagelkerke’s method) of the variance in the likelihood of ICU transfer, accurately classifying 73% of cases (Table 5).

The predictors significant in predicting the probability of hospitalization for 10 days or more are presented in Table 6. Through multivariate logistic regression, one predictor stood out, HFNC upon admission, which was entirely statistically significant (χ^2^ = 12.6, *p* < 0.001), explaining between 12% (using Cox and Snell’s method) and 17% (using Nagelkerke’s method) of the variance in hospitalization for 10 days or more, accurately classifying 69% of cases (Table 6).

**Figure 2:**
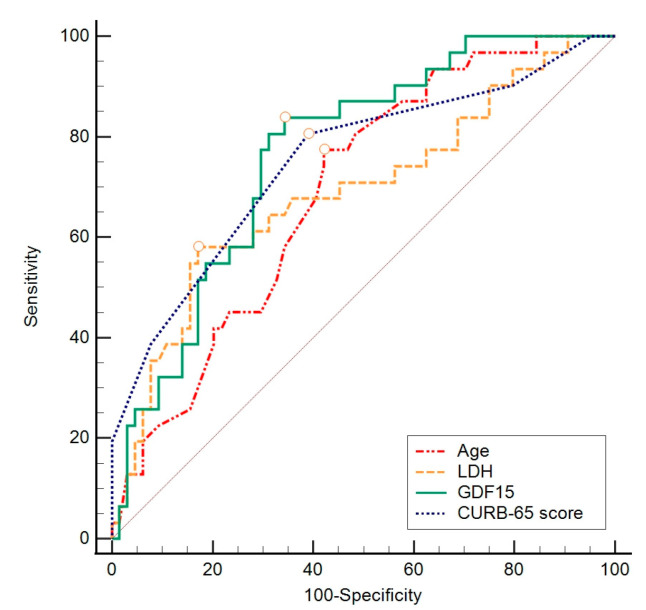
ROC analysis of sensitivity, specificity, and threshold values for observed variables concerning negative outcomes of the disease. LDH—lactate dehydrogenase; GDF-15—growth differentiation factor 15.

**Figure 3:**
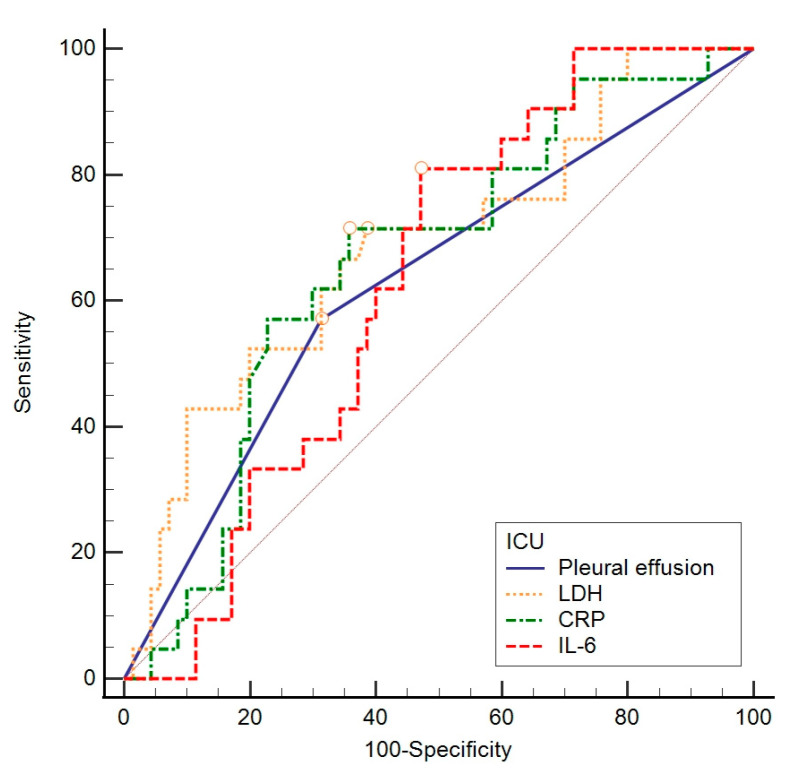
ROC analysis of sensitivity, specificity, and threshold values for observed values with respect to the ICU transfer. ICU—intensive care unit; LDH—lactate dehydrogenase; CRP—C-reactive protein; IL-6—interleukin 6.

**Figure 4:**
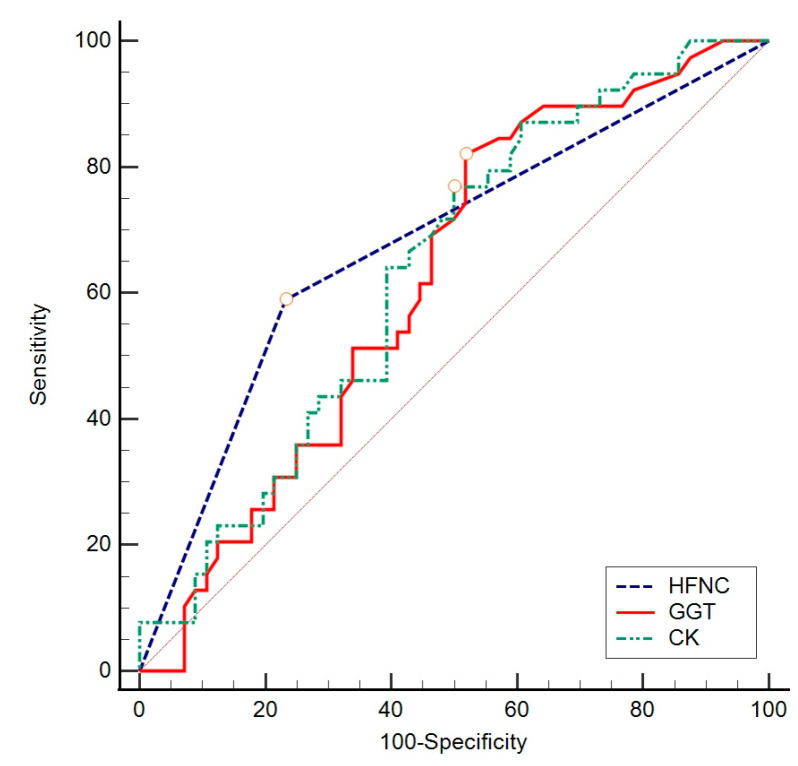
ROC analysis of sensitivity, specificity, and threshold values for observed values with respect to hospitalization longer than or equal to 10 days. HFNC—high-flow nasal cannula; GGT—gamma-glutamyltransferase; CK—creatine kinase.

The ROC curve method was chosen to assess the difference in individual indicators between groups concerning a negative outcome, ICU admission, and length of hospitalization, based on specificity and sensitivity. To assess the value of individual parameters found to significantly contribute to previous analyses, the ROC curve calculation method was employed. This involved gradually changing the values that distinguished subjects concerning the outcome, creating the ROC curve objectively (Figure 2, Figure 3 and Figure 4) to determine which value best distinguished the compared groups. In these data, considering a negative outcome, significant diagnostic indicators include the age of the subjects, CURB-65 score, LDH, and GDF-15. The diagnostic indicator with the largest area under the curve is represented by GDF-15, although the other values presented in Table 7 also serve as significant diagnostic indicators.

For ICU admission, significant diagnostic indicators are LDH, CRP, and IL-6 (Table 8). Furthermore, considering the length of hospitalization, significant diagnostic indicators are CK, GGT, and oxygenation with an HFNC (Table 9).

## 4. Discussion

This study evaluated the predictive and diagnostic value of routine laboratory parameters, clinical scores (CURB-65 and qSOFA scores), and GDF-15 in hospitalized COVID-19 patients. It is worth noting that it showed an association between elevated levels of GDF-15 and negative outcome in COVID-19 patients. Furthermore, certain measured laboratory parameters, clinical scores, and interventions showed associations with mortality, admission to the ICU, and the prolonged hospitalization of the mentioned group of patients. 

The observed patient cohort primarily consisted of elderly individuals who were unvaccinated and had not experienced COVID-19 before. Additionally, a significant proportion of the patients had at least one comorbidity, with nearly all of them complicated by pneumonia. It is worth noting that these findings align with expectations given that the study exclusively focused on hospitalized patients and are in line with the previous research, which has consistently identified an older age and the presence of comorbidities as significant risk factors for hospitalization among COVID-19 patients. Moreover, vaccination has been reported in prior studies to effectively reduce the risk of hospitalization in individuals afflicted with COVID-19 [27,28].

### 4.1. Findings Regarding GDF-15

**Figure 5:**
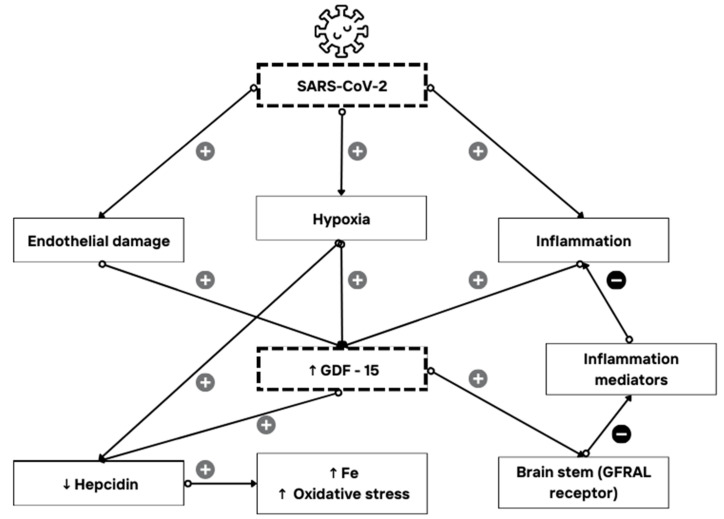
Proposed mechanism of SARS-CoV-2, GDF-15, and iron metabolism interaction. SARS-CoV-2 causes endothelial damage, tissue hypoxia, and inflammation due to the direct impact of the virus, coagulopathy, complement activation, and hypovolemia in severe forms of COVID-19. Endothelial damage, hypoxia, and inflammation have all been previously reported to result in higher expression of GDF-15 and an increase in its levels. Both GDF-15 and hypoxia have been shown to lower hepcidin levels, leading to the loss of the normal role of hepcidin—an acute-phase reactant—in reducing serum iron during an infection. This disruption leads to impaired iron homeostasis, elevated serum iron values, and oxidative stress. Additionally, GDF-15 directly reduces the release of various inflammation mediators by binding to the GDNF family receptor α–like (GFRAL) in the brainstem, thereby decreasing the extent of inflammation. These findings suggest a possible dual role of GDF-15 in acute inflammation: a protective one through GFRAL receptors and a non-protective role through hepcidin regulation [5,12,30,31,32,33]. However, further research is needed.

In this research, we assessed not only mortality but also ICU admission and the length of hospitalization, as all these findings can indicate the severity of COVID-19. The study aimed to introduce a possible novel biomarker for predicting COVID-19 severity, GDF-15. Generally speaking, the median values of GDF-15 in all the observed groups were higher than those expected in healthy individuals according to Doerstling et al. [29], implying the effect of SARS-CoV-2 on the GDF-15 levels in hospitalized patients. GDF-15 was more highly expressed in the non-survivor group compared to the survivor group, with a median value of 5762.0 pg/mL. The ROC analysis marked GDF-15 as a diagnostic indicator of a negative outcome with moderate specificity and sensitivity. Nevertheless, there was no statistically significant relationship between the GDF-15 levels and ICU admission and length of hospitalization. It has already been shown that GDF-15 levels rise in stress states, and its expression can be induced by hypoxia and endothelial damage [1,8,9,12], as well as aging [6,7]. SARS-CoV-2 has affinity for causing such states—tissue hypoxia as part of pneumonia-caused respiratory failure and endothelial damage due to direct impact of virus, coagulopathy, complement activation, and hypovolemia in severe forms of COVID-19 [12,30,31,32]. When considering the above, it is reasonable to anticipate elevated levels of GDF-15 in patients with severe manifestations of COVID-19, as detailed in the hypothesized mechanism explained in Figure 5. Our findings align with this expectation, supporting similar observations made by other authors [19,20,21]. However, this pilot study examines the associations between GDF-15 and clinical outcomes; it does not directly demonstrate causality. Furthermore, the specified cutoff value of 3528 pg/mL, determined through the ROC analysis, can assist clinicians in assessing the mortality risk in hospitalized COVID-19 patients when observing this parameter, thereby enabling timely and appropriate treatment to reduce negative outcomes.

This is not to neglect that higher GDF-15 levels were associated with a higher CURB-65 score, as well as a higher qSOFA score, older age, and elevated CRP, PCT, and IL-6 levels. Our results suggest that older age, a higher CURB-65 score, raised CRP values, and elevated IL-6 levels could predict a negative outcome. It is also recognized that an age greater than 74 years and a CURB-65 score higher than 2 serve as possible diagnostic indicators of a negative outcome. These findings make us believe that GDF-15 could potentially provide stronger evidence as a biomarker for severe COVID-19 in a larger cohort of patients.

The found association of GDF-15 with the PCT levels of non-survivors and ICU patients in this cohort also introduces the need for further research into GDF-15’s possible connections with other, more studied inflammatory markers. Considering the speculated mechanism of GDF-15’s interaction with SARS-CoV-2 and endothelial tissue, further studies should concentrate on the association between GDF-15 and other inflammatory markers elevated in endothelial dysfunction, such as platelet factor 4 (PF-4, CXCL4) [34,35], which has recently been studied in COVID-19 patients [36,37]. The findings of such studies could help us better understand the pathophysiological mechanisms of GDF-15 release in COVID-19 and in general.

Given that increases in GDF-15 values do not always align with those of other inflammatory markers, we should also consider research aimed at investigating how early in the course of infectious or inflammatory diseases GDF-15 levels rise. Understanding the precise dynamics of GDF-15 levels throughout disease progression could help us confirm or dismiss its role as an early diagnostic biomarker. It is plausible that its values peak earlier or later in the disease course compared to established acute-phase reactants.

Since there is a receptor for GDF-15 in the brainstem, it would be interesting to observe its levels in patients with COVID-19 and neurological comorbidity. An umbrella review of 9,228,588 COVID-19 patients by Jong Mi Park et al. showed an increased mortality risk in patients with certain neurological conditions [38]. Further exploration of the mechanisms behind such events, which could be linked to the inflammatory process and biomarkers like GDF-15, is warranted. Another interesting field for further exploration of GDF-15 is in patients with low seropositivity after COVID-19 vaccination, as described in a systematic review by Kyuyeon Cho et al. Low seropositivity is often found in patients with weakened immunity due to comorbidities, and an adequate biomarker to differentiate such patients in the general population has yet to be found [39]. Such a biomarker could be helpful in distinguishing these patients and providing the appropriate care.

### 4.2. Highlighted Findings on Other Laboratory Parameters

The study demonstrates that various routinely determined parameters can be beneficial during the initial evaluation of patients concerning each observed criterion—clinical outcomes, ICU admission, and length of stay. 

When considering the population observed in this research, both higher CRP values and higher LDH values were expressed in the less favourable groups—non-survivors, ICU-admitted patients, and those hospitalized for 10 or more days. Additionally, LDH stood out as predictor of negative outcomes and of transfer to the ICU, as well as a diagnostic indicator of a negative outcome with a cutoff value of 395 U/L and of transfer to the ICU with a cutoff value of 404 U/L. C-reactive protein is a known inflammatory marker, and its levels are usually higher in acute inflammation, especially with a larger extent of inflammation [40]. The results obtained in this study lead to the conclusion that higher CRP levels are unfavorable in hospitalized COVID-19 patients. LDH is recognized as a marker of tissue damage and is elevated in a large number of diseases, but our findings also suggest that LDH levels should be considered when evaluating COVID-19 patients. Such results for CRP and LDH are also supported by findings from other authors [41,42]. 

Both of the aforementioned biomarkers, as well as others proven statistically significant in this study, are common in clinical use, widely available, and usually cost-effective. 

### 4.3. The Study’s Limitations

This study is primarily a pilot study, as it involved a limited number of patients, with a high proportion having cardiovascular comorbidities. It is a single-centre study conducted in one country, and the generalizability of our results is unknown. Therefore, further research in this field is warranted. Nonetheless, our study can certainly serve as a foundation for such research. The main limitation of this study is the sample size, which may explain the absence of statistically significant differences in the GDF-15 concentrations between the groups of ICU and non-ICU patients and between those hospitalized for less than ten days and those hospitalized for ten or more days. Despite this limitation, the median values of GDF-15 were higher in the ICU group of patients and in those hospitalized for ten or more days. It is possible to presume that studies on a larger sample of patients could potentially strengthen GDF-15’s diagnostic significance in survivors and non-survivors, as well as possibly show its significance in ICU and non-ICU patients or patients with prolonged hospitalization. Such studies are yet to be conducted to either confirm or refute our findings. 

This research also faces confounding bias, as some of the observed patients had comorbidities that could theoretically have affected their GDF-15 levels, such as adiposity or chronic diseases. Omitting comorbidities in the study was not feasible, as individuals with COVID-19 requiring hospitalization tended to have such medical conditions.

Another limitation of this study is the lack of observation of the patients after hospitalization. Our study lacks an assessment of downstream morbidity and mortality. More precisely, GDF-15 levels could also be measured after the clinical resolution of COVID-19, and it remains to be determined whether the levels of GDF-15 will decrease to their basal values. Such results could help better determine the impact of comorbidities (confounding bias) on GDF-15 levels and shed light on GDF-15 levels in long COVID-19 compared to patients who achieve full recovery.

## 5. Conclusions

Considering the obtained results, our hypothesis is partially accepted, and further research is necessary.

To our knowledge, the reports on GDF-15 expression during COVID-19 are limited and somewhat contradictory. Some studies have been conducted on specific patient groups, such as patients on hemodialysis or those with chronic kidney disease, where underlying diseases could affect the GDF-15 levels [14,43]. Most of the previously performed studies support evidence of higher GDF-15 levels in COVID-19 patients requiring hospitalization and those with a more severe form of the disease [15,16,17,18,19,20,21,22], while others have provided data supporting lower levels of GDF-15 in hospitalized COVID-19 patients or no significant difference between hospitalized and discharged patients, nor between SARS-CoV-2-positive and -negative patients [18,23,24]. Our findings align with the majority of studies conducted so far, indicating a higher expression of GDF-15 at the moment of hospitalization in patients with a negative outcome of COVID-19. As far as we are aware, this is the first study examining GDF-15 expression in relation to three key parameters in assessing disease severity: clinical outcomes, ICU admission, and the duration of hospitalization. Since studies are scarce, it is imperative to continue research on this topic with larger patient cohorts to fully understand the potential of GDF-15 as a biomarker of COVID-19 severity and to recognize its pathophysiological pathways in this disease, as well as in others. Questions regarding the dynamics of GDF-15 elevation and its time interval for chronic diseases, pregnancy, adiposity, and other prolonged clinical states, as well as the same questions for acute diseases such as COVID-19 and sepsis, remain to be pondered. Also, the expected levels of GDF-15 and cutoff values in those states require further study.

In conclusion, this study reaffirms the significance of commonly used laboratory parameters and clinical scores in the evaluation of COVID-19, but it also introduces the potential for a new diagnostic approach and research concerning GDF-15 levels in this widespread disease.

## Figures and Tables

**Table 1:** Baseline demographic and clinical characteristics of participants.

Sex [*n* (%)]	
Male	41 (43)
Female	54 (57)
Age [Median (range)]	76 (22–93)
Length of hospitalization [Median (IQR)]	9 (6–13)
Mean arterial pressure—MAP (mmHg)	83.3 (76.7–93.3)
Vaccinated [*n* (%)]	34 (36)
Comorbidities [*n* (%)]	
Type 2 diabetes mellitus	21 (22)
Hypertension	68 (72)
Cardiomyopathy	21 (22)
Atrial fibrillation	9 (10)
Lung disease	10 (11)
Chronic kidney disease	4 (4)
Clinical symptoms [*n* (%)]	
General symptoms	
Fever	71 (75)
Cephalea	5 (5)
Chills/shivering	19 (20)
General weakness	63 (66)
Nausea	15 (16)
Myalgia or arthralgia	21 (22)
Confusion	15 (16)
Respiratory symptoms	
Cough	81 (85)
Sore throat	5 (5)
Chest pain	28 (30)
Hemoptysis	3 (3)
Dyspnea (anamnestic)	60 (63)
Dyspnea (at examination)	73 (77)
Gastrointestinal symptoms	
Diarrhea	26 (27)
Vomiting	6 (6)
Skin changes	2 (2)
Complications [*n*(%)]	
Pulmonary complications	
Pneumonia	91 (96)
Unilateral involvement	10/91 (11)
Bilateral involvement	81/91 (89)
Pulmonary embolism	1 (1)
Pleural effusion	36 (38)
Oxygenation (nasal catheter or mask)	89 (94)
Oxygenation (high-flow nasal cannula—HFNC)	36 (38)
Invasive mechanical ventilation (IMV)	20 (21)

IQR—interquartile range.

**Table 2:** Baseline characteristics of the participants according to the studied outcomes.

	Clinical Outcome		*p* ^3^	ICU Admission		*p* ^3^	Length of Hospitalization		*p* ^3^
	Survivors (*n* = 64)	Non-Survivors (*n* = 31)		Non-ICU (*n* = 71)	ICU (*n* = 24)		Less than 10 Days (*n* = 52)	10 and More Days (*n* = 43)	
Demographic data									
Female, *n* (%)	36 (56)	18 (58)	0.870 ^1^	41 (58)	13 (54)	0.760 ^1^	31 (59)	23 (54)	0.550 ^1^
Age in years, median (range)	71 (22–90)	79 (56–93)	0.003	74 (22–93)	77 (56–89)	0.400	77 (22–93)	76 (33–89)	0.550
Vaccinated, *n* (%)	25 (39)	9 (29)	0.340^1^	28 (39)	65 (25)	0.200 ^1^	22 (42)	12 (28)	0.150 ^1^
Length of hospitalization in days, median (IQR)	8 (6–13)	11 (7–15)	0.090	8 (6–12)	13 (8–15)	0.002	7 (5–8)	14 (12–16)	<0.001
MAP in mmHg, median (IQR)	83.3 (80–93.3)	83.3 (73.3–86.7)	0.120	83.3 (73.3–90)	85 (78.3–83.3)	0.240	83.3 (73.3–89.6)	83.3 (80–93.3)	0.340
Comorbidity, *n* (%)									
Type 2 diabetes mellitus	15 (23)	6 (19)	0.650 ^1^	15 (21)	6 (25)	0.690 ^1^	13 (25)	8 (19)	0.460 ^1^
Hypertension	42 (66)	26 (84)	0.070 ^1^	50 (70)	18 (75)	0.670 ^1^	36 (69)	32 (74)	0.580 ^1^
Cardiomyopathy	10 (16)	11 (36)	0.030 ^1^	13 (18)	8 (33)	0.130 ^1^	10 (19)	11 (26)	0.460 ^1^
Atrial fibrillation	4 (6)	5 (16)	0.15 ^2^	7 (10)	2 (8)	>0.990 ^2^	4 (8)	5 (12)	0.730 ^2^
Lung disease	6 (9)	4 (13)	0.720 ^1^	7 (10)	3 (13)	0.710 ^1^	5 (10)	5 (12)	0.750 ^1^
Chronic kidney disease	2 (3)	2 (7)	0.600 ^2^	3 (4)	1 (4)	>0.990 ^2^	3 (6)	1 (2)	0.620 ^2^
Clinical scores, median (IQR)									
qSOFA score	1 (1–1)	1 (1–2)	0.010	1 (1–2)	1 (1–2)	0.760	1 (1–2)	1 (1–1)	0.670
CURB-65 score	2 (2–3)	3 (3–4)	<0.001	1 (2–3)	2 (2–4)	0.200	3 (2–3)	3 (2–3)	0.580
Laboratory findings, median (IQR)									
WBC (×10^9^/L)	6.6 (5.3–9.4)	6.8 (4.6–11.9)	0.990	6.4 (5.1–10.1)	7.5 (4.8–10.1)	0.870	7.1 (5.1–10.5)	6.3 (5.1–9.3)	0.570
RBC (×10^12^/L)	4.5 (4–5)	4.3 (4–4.9)	0.380	4.46 (4–5)	4.48 (4–4.9)	0.840	4.39 (3.9–4.9)	4.64 (4.1–5)	0.060
Hb (g/L)	136 (119–146)	131 (120–146)	0.850	135 (119–144)	135 (122–1501)	0.470	130 (116–143)	138 (125–150)	0.050
Hct (L/L)	0.4 (0.4–0.4)	0.4 (0.4–0.4)	0.810	0.4 (0.4–0.4)	0.41 (0.4–0.4)	0.340	0.39 (0.4–0.4)	0.41 (0.4–0.4)	0.040
MCV fL)	87.8 (84.6–91.8)	89.9 (87–93.7)	0.110	88.2 (84.5–92.6)	89.05 (87–93.4)	0.330	88.9 (86.1–92.9)	87.4 (84.3–92.3)	0.220
MCHC (g/L)	334 (327–342)	331 (320–338)	0.040	333 (326–342)	332 (321–342)	0.410	334 (325–341)	333 (326–342)	0.920
RDW-CV (%)	13.3 (12.9–14.1)	14.3 (13.4–15.9)	0.002	13.4 (13.1–14.6)	13.95 (13–15.2)	0.330	13.4 (13–14.7)	13.7 (13.1–14.6)	0.620
Platelets (×10^9^/L)	213 (155–273)	195 (138–224)	0.220	210 (148–281)	197 (159–222)	0.390	204 (159–307)	195 (138–251)	0.130
Neutrophils (%)	76 (68–86)	79 (73–87)	0.200	76 (68–86)	80 (74–88)	0.110	76 (68–87)	78 (70–86)	0.320
Lymphocytes (%)	15 (8–22)	12 (7–17)	0.190	15 (8–21)	12 (6–16)	0.120	15 (8–23)	12 (7–17)	0.170
Band cells (%)	0 (0–0)	0 (0–0)	0.080	0 (0–0)	0 (0–0)	0.930	0 (0–0)	0 (0–0)	0.230
Monocytes (%)	8 (5–9)	6 (4–9)	0.170	8 (5–9)	6 (4–9)	0.270	8 (5–9)	7 (5–10)	0.590
Basophils (%)	0 (0–0)	0 (0–0)	0.110	0 (0–0)	0 (0–0)	0.180	0 (0–0)	0 (0–0)	0.240
Eosinophils (%)	0 (0–7)	0 (0–0)	0.030	0 (0–0)	0 (0–0)	0.070	0 (0–7)	0 (0–1)	0.410
PT (INR)	0.9 (0.9–1)	1 (0.9–1)	0.160	0.94 (0.9–1)	0.97 (0.9–1)	0.950	0.95 (0.9–1)	0.96 (0.9–1)	0.790
Fbg (g/L)	6.4 (5.7–7.9)	6.1 (5.4–8.6)	0.830	6.4 (5.4–7.9)	6.4 (5.6–9.5)	0.450	6.35 (5.4–7.4)	6.6 (5.6–9.5)	0.140
APTT (1)	0.8 (0.8–0.9)	0.9 (0.8–1)	0.110	0.85 (0.8–0.9)	0.82 (0.8–0.9)	0.560	0.85 (0.8–0.9)	0.82 (0.8–0.9)	0.400
D-dimers (μg/L FEU)	1281 (799–2173)	1514 (1124–3117)	0.330	1335 (808–2120)	1438 (1037–3356)	0.430	1286 (816–2110)	1506 (840–2685)	0.710
AT-3 (1)	1.1 (1–1.3)	1.1 (1–1.2)	0.060	1.1 (1–1.3)	1.1 (0.9–1.2)	0.200	1.1 (1–1.2)	1.1 (1–1.3)	0.650
AST (U/L)	44 (31–66)	57 (43–98)	0.003	44 (34–66)	67 (44–98)	0.008	45 (29–68)	50 (41–86)	0.060
ALT (U/L)	34 (19–51)	36 (23–51)	0.510	31 (16–50)	39 (30–53)	0.100	31 (17–52)	36 (23–50)	0.330
ALP (U/L)	68 (56–89)	57 (51–75)	0.180	63 (53–89)	63 (50–75)	0.240	61 (55–86)	64 (50–89)	0.570
GGT (U/L)	41 (24–101)	37 (20–96)	0.630	39 (22–90)	38 (23–111)	0.720	30 (17–86)	50 (30–116)	0.030
CK (U/L)	82 (50–228)	142 (74–386)	0.030	83 (55–223)	112 (73–430)	0.080	76 (49–233)	107 (78–288)	0.030
LDH (U/L)	309 (255–382)	405 (278–491)	0.003	314 (253–392)	405 (298–492)	0.005	296 (252–400)	365 (282–432)	0.030
Urea (mmol/L)	6.9 (5.5–9.4)	9.1 (6.9–16)	0.010	7.1 (5.6–10.3)	7.5 (6.2–11.8)	0.390	7.1 (6–10.7)	7.4 (5.5–10.7)	0.850
Creatinine (μmol/L)	80 (65–96)	93 (79–152)	0.004	83 (66–107)	90 (78–114)	0.160	82 (65–108)	86 (72–116)	0.340
Albumin (g/L)	34.7 (31.2–36.8)	32.2 (30.2–37.2)	0.220	34.45 (30.6–36.6)	33.8 (31.4–37.7)	0.510	34.8 (31.1–37.1)	33.6 (30.5–36.3)	0.420
CRP (mg/L)	64 (30.5–110.9)	116.6 (73.3–158.4)	0.002	71.9 (30.9–114.2)	116.8 (60.9–152.8)	0.010	72.6 (30.1–109.1)	101.7 (48.3–152.1)	0.020
PCT (μg/L)	0.1 (0.1–0.2)	0.2 (0.1–0.4)	0.002	0.13 (0.1–0.3)	0.18 (0.1–0.3)	0.150	0.14 (0.1–0.3)	0.17 (0.1–0.3)	0.310
hsTnI (ng/L)	16.8 (8.9–35.5)	29.8 (21.4–64.8)	0.020	24.6 (9.9–57.7)	26.2 (12.6–53.1)	0.960	21.4 (9.8–66)	26.7 (12.3–37.9)	0.550
IL-6 (ng/L)	50.4 (24.5–115.6)	96.9 (58.6–179.5)	0.003	55.6 (26.2–121.2)	76.3 (58.7–154)	0.080	53 (20.5–124)	70.6 (36–136.3)	0.16
Ferritin (μg/I)	582.8 (200–1036.6)	559.8 (234–1275.4)	0.700	528.3 (199.5–1071.7)	594.2 (291.1–1185.9)	0.650	422.8 (191.8–1101.3)	653.5 (361–1070.8)	0.260
Na (mmol/L)	138 (136–140)	138 (137–141)	0.350	138 (136–141)	138 (137–141)	0.750	139 (136–141)	138 (136–141)	0.400
K (mmol/L)	4.1 (3.7–4.4)	4.2 (3.7–4.4)	0.640	4.1 (3.7–4.4)	4.2 (3.8–4.4)	0.450	4.2 (3.7–4.4)	4.1 (3.7–4.4)	0.990
Cl (mmol/L)	101 (96–103)	101 (99–104)	0.360	100 (96–103)	101 (99–104)	0.450	102 (98–104)	100 (96–102)	0.060
Glucose (mmol/L)	7.1 (6.1–9)	7.2 (6–9.8)	0.970	6.9 (6.1–8.6)	7.4 (6.1–10.2)	0.280	6.8 (6–8.5)	7.4 (6.2–9.9)	0.150
GDF-15 (pg/mL)	3078.0 (1671.5–5041.3)	5762.0 (4213.0–10,795)	<0.001	3298 (2144–6282)	4845.5 (3039–6700)	0.080	3517 (1957.3–6419)	4143 (2842–5762)	0.590

^1^ Chi-square test; ^2^ Fisher’s exact test; ^3^ Mann–Whitney U test, IQR—interquartile range; ICU—intensive care unit; WBC—white blood cells; RBC—red blood cells; Hb—hemoglobin; Hct—hematocrit, MCV—mean cell volume; MCHC—mean corpuscular hemoglobin concentration; RDW-CV—red blood cell distribution width—coefficient of variation; PT—prothrombin time; INR—international normalized ratio; Fbg—fibrinogen; APTT—activated partial thromboplastin time; AT-3—antithrombin 3; AST—aspartate aminotransferase; ALT—alanine aminotransferase; ALP—alkaline phosphatase; GGT—gamma-glutamyltransferase; CK—creatine kinase; LDH—lactate dehydrogenase; CRP—C-reactive protein; PCT—procalcitonin; hsTnI—high-sensitivity troponin I; IL-6—interleukin 6; Na—sodium; K—potassium; Cl—chloride; GDF-15—growth differentiation factor 15.

**Table 3:** The association of GDF-15 with age and qSOFA and CURB-65 scores, as well as inflammatory values upon admission, in relation to the outcome and transfer to the ICU in the group of all patients.

	Spearman’s Rank Correlation Coefficient Rho (*p* Value) GDF-15				
	All Patients	Clinical Outcome		ICU Admission	
		Survivors	Non-Survivors	Non-ICU	ICU
Age	0.442 (<0.001)	0.479 (<0.001)	0.152 (0.420)	0.541 (<0.001)	−0.011 (0.960)
qSOFA score	0.307 (0.003)	0.055 (0.660)	0.644 (<0.001)	0.248 (0.040)	0.499 (0.010)
CURB-65 score	0.537 (<0.001)	0.427 (<0.001)	0.541 (0.002)	0.534 (<0.001)	0.448 (0.030)
Inflammatory markers					
CRP	0.337 (0.001)	0.319 (0.010)	0.042 (0.820)	0.380 (0.001)	−0.012 (0.960)
PCT	0.586 (<0.001)	0.482 (<0.001)	0.619 (<0.001)	0.541 (<0.001)	0.656 (0.001)
IL-6	0.461 (<0.001)	0.401 (0.001)	0.424 (0.030)	0.520 (<0.001)	0.106 (0.650)

ICU—intensive care unit; CRP—C-reactive protein; PCT—procalcitonin; IL-6—interleukin 6.

**Table 4:** Prediction of the probability of a negative outcome (bivariate and multivariate regression).

Negative Outcome	β	Wald	*p* Value	Odds Ratio	95% CI
Bivariate regression					
Age	0.07	8.48	0.004	1.07	1.02 to 1.12
Cardiomyopathy	1.09	4.57	0.030	2.97	1.09 to 8.6
CURB-65 score	0.99	14.6	<0.001	2.7	1.62 to 4.5
MCHC	−0.05	6.32	0.010	0.95	0.91 to 0.98
RDW-CV	0.25	4.4	0.040	1.29	1.02 to 1.64
AST	0.02	6.95	0.008	1.02	1.005 to 1.03
CK	0.001	4.05	0.040	1.001	1.00 to 1.0003
LDH	0.005	7.99	0.005	1.005	1.002 to 1.009
Urea	0.12	7.27	0.007	1.13	1.03 to 1.23
Creatinine	0.01	7.33	0.007	1.01	1.004 to 1.03
CRP	0.008	6.54	0.010	1.01	1.002 to 1.01
PCT	0.96	3.36	0.070	2.60	0.94 to 7.22
hsTnI	−0.001	0.01	0.910	0.99	0.99 to 1.001
IL-6	0.002	3.87	0.040	1.002	1.00 to 1.005
GDF-15	0.0002	7.99	0.005	1.0002	1.0001 to 1.0003
Multivariate regression					
CURB-65 score	0.99	11.21	<0.001	2.55	1.48 to 4.42
LDH	0.005	5.05	0.020	1.01	1.001 to 1.009
Constant	−5.31	18.01	<0.001		

β—regression coefficient; MCHC—mean corpuscular hemoglobin concentration; RDW-CV—red blood cell distribution width—coefficient of variation; AST—aspartate aminotransferase; CK—creatine kinase; LDH—lactate dehydrogenase; CRP—C-reactive protein; PCT—procalcitonin; hsTnI—high-sensitivity troponin I; IL-6—interleukin 6; GDF-15—growth differentiation factor 15.

**Table 5:** Prediction of the probability of ICU admission (bivariate and multivariate regression).

ICU Admission	β	Wald	*p* Value	Odds Ratio	95% CI
Bivariate regression					
Age	0.03	1.91	0.170	1.03	0.98 to 1.07
Cardiomyopathy	0.80	2.29	0.130	2.23	0.79 to 6.31
CURB-65 score	0.23	1.19	0.270	1.26	0.84 to 1.89
Pleural effusion	1.14	5.45	0.020	3.12	1.20 to 8.10
AST	0.005	1.47	0.230	1.01	0.99 to 1.01
LDH	0.004	4.65	0.030	1.004	1.0003 to 1.007
CRP	0.005	3.37	0.070	1.005	0.99 to 1.01
IL-6	−0.001	0.39	0.530	0.99	0.99 to 1.002
GDF-15	0	0.79	0.370	1.00	1.00 to 1.0001
Multivariate regression					
LDH	0.004	4.33	0.030	1.004	1.0003 to 1.007
Constant	−2.55	12.8	<0.001		

β—regression coefficient; ICU—intensive care unit; AST—aspartate aminotransferase; LDH—lactate dehydrogenase; CRP—C-reactive protein; IL-6—interleukin 6; GDF-15—growth differentiation factor 15.

**Table 6:** Prediction of the probability of hospitalization for 10 days or more (bivariate and multivariate regression).

Length of Hospitalization ≥ 10 Days	β	Wald	*p* Value	Odds Ratio	95% CI
Bivariate regression					
Age	0.007	0.20	0.650	1.01	0.97 to 1.03
Bilateral pneumonia	1.39	4.12	0.040	4.0	1.05 to 15.3
Pleural effusion	0.86	3.93	0.040	2.35	1.01 to 5.49
High-flow nasal cannula (HFNC)	1.44	10.2	0.001	4.21	1.74 to 10.17
IMV	1.62	8.07	0.005	5.04	1.65 to 15.36
GGT	0.002	0.32	0.570	1.002	0.99 to 1.01
CK	0.001	2.09	0.150	1.001	0.99 to 1.002
LDH	0.003	2.52	0.110	1.003	0.99 to 1.01
CRP	0.007	5.25	0.020	1.01	1.001 to 1.01
GDF-15	−0.0003	0.49	0.480	1.00	0.99 to 1.0001
Multivariate regression					
High-flow nasal cannula (HFNC)	1.56	11.8	<0.001	4.75	1.95 to 11.6
Constant	−0.99	11.4	<0.001		

β—regression coefficient; IMV—invasive mechanical ventilation; GGT—gamma-glutamyl transferase; CK—creatine kinase; LDH—lactate dehydrogenase; CRP—C-reactive protein; GDF-15—growth differentiation factor 15.

**Table 7:** Diagnostic value of patient’s age, GDF-15, CURB-65, and LDH (ROC analysis) with respect to a negative outcome.

	GDF-15						
	AUC	95% CI	Sensitivity	Specificity	Cut Off	Youden Index	*p* Value
GDF-15 (pg/mL)	0.767	0.669–0.848	83.9	65.6	>3528	0.50	<0.001
Age (years)	0.691	0.588–0.782	77.4	57.8	>74	0.35	<0.001
CURB-65 score	0.752	0.653–0.835	80.6	60.9	>2	0.42	<0.001
LDH (U/L)	0.691	0.587–0.781	58.1	82.8	>395	0.41	0.002

AUC—area under the curve; GDF-15—growth differentiation factor 15; LDH—lactate dehydrogenase.

**Table 8:** Diagnostic value of patient’s LDH, CRP, and IL-6 (ROC analysis) with respect to an ICU admission.

	AUC	95% CI	Sensitivity	Specificity	Cut Off	Youden Index	*p* Value
Pleural effusion	0.637	0.532–0.733	58.3	69.0	>0	0.27	0.020
LDH (U/L)	0.694	0.591–0.784	54.2	80.3	>404	0.34	0.002
CRP (mg/L)	0.676	0.572–0.768	70.8	64.8	>95.9	0.36	0.004
IL-6 (ng/L)	0.629	0.521–0.728	81.0	52.9	>58.5	0.34	0.030

AUC—area under the curve: ICU—intensive care unit; LDH—lactate dehydrogenase; CRP—C-reactive protein; IL-6—interleukin 6.

**Table 9:** Diagnostic value of patient’s need for HFNC and levels of GGT and CK (ROC analysis) with respect to hospitalization for 10 days or more.

	AUC	95% CI	Sensitivity	Specificity	Cut Off	Youden Index	*p* Value
High-flow nasal cannula (HFNC)	0.679	0.575–0.771	59.0	76.8	>0	0.36	<0.001
GGT (U/L)	0.621	0.516–0.719	82.1	48.2	>28	0.30	0.030
CK (U/L)	0.635	0.530–0.731	76.9	50.0	>76	0.27	0.020

AUC—area under the curve, GGT—gamma-glutamyltransferase; CK—creatine kinase.

## Data Availability

The data that support the findings of this study are available from the corresponding author, S.M., upon reasonable request.
